# *‘dstidyverse’: An Implementation of* *TidyverseWithin the DataSHIELD* *Ecosystem*

**DOI:** 10.12688/f1000research.164345.1

**Published:** 2025-06-20

**Authors:** Tim Cadman, Mariska Slofstra, Demetris Avraam, Eleanor Hyde, Niels Kikkert, Marije van der Geest, Dick Postma, Ruben Veenstra, Stuart Wheater, Erik Zwart, Morris Swertz

**Affiliations:** 1 Department of Genetics, Genomics Coordination Center, University Medical Centre, Groningen, The Netherlands; 2 Barcelona Institute for Global Health (ISGlobal), Barcelona, Spain; 3 Department of Public Health, University of Copenhagen, Øster Farimagsgade, Copenhagen, Denmark; 4 Newcastle Helix, Urban Science Building, Newcastle upon Tyne, Arjuna Technologies, Newcastle, UK

**Keywords:** datashield, federated analysis, R, tidyverse, data manipulation

## Abstract

**Background:**

DataSHIELD is a mature, R-based federated learning platform that enables multi-site analysis without sharing individual participant data. While DataSHIELD includes many packages for data analysis, it lacks user-friendly data manipulation tools.

**Methods:**

To address this gap, we developed **dsTidyverse**, an implementation of selected functions from the popular Tidyverse package within the DataSHIELD client-server architecture. Disclosure checks were implemented to prevent individual-level data leakage.

**Results:**

This package provides functionality for selecting, renaming, and creating columns; conditional recoding; combining data frames by rows or columns; filtering and arranging rows; grouping and ungrouping data; and converting data frames to tibbles. Through examples, we demonstrate how **dsTidyverse** simplifies common data manipulation tasks within DataSHIELD.

**Conclusions:**

By providing additional data manipulation functionality, **dsTidyverse** improves the user experience and analytical efficiency within DataSHIELD. The package is open-source and freely available on CRAN and GitHub, and welcomes further development: https://github.com/molgenis/ds-tidyverse.

## 1. Introduction

While the analysis of single data sources is a core part of epidemiological research, incorporating data from multiple sources has a number of advantages. These include increased statistical power to detect rare disease outcomes and the opportunity to replicate studies in different populations ( Pinot de Moira et al. (2021) Cadman et al. (2024)). Historically, the analysis of multiple data sources has been conducted by either (i) data transfer or (ii) each partner conducting analyses separately and sharing summary statistics. Although both approaches are effective in many situations, they have drawbacks. The physical transfer of data can be restricted by data protection legislation and local data management policies, while requiring each partner to conduct parallel analyses can be time inefficient and inflexible ( Knoppers et al. (2011)).

A promising alternative is federated (remote) analysis which does not share individual-level data. Federated analysis allows one researcher to conduct all analyses flexibly, while allowing control of the data to remain with the data owner ( Doiron et al. (2013)). One mature implementation of federated analysis is the open-source R-based platform DataSHIELD ( Gaye et al. (2014)). DataSHIELD is based on a client-server architecture. In a multisite setting, individual study participants’ data are stored on the server of each data source, often protected by a firewall. The data from each site are not directly viewable or accessible to the analyst and cannot be copied or transferred. On the client side, the researcher has access to several DataSHIELD-specific R packages. Using the functions from these packages, the researcher issues analysis commands that are then sent to each server. There are two types of DataSHIELD functions: (i) assign-type functions, which create a new object on the server (e.g., recoding a variable), and (ii) aggregate-type functions, which return summary statistics to the researcher (e.g., means, standard deviations and model parameters). These commands are evaluated on each server, and automated checks are performed to ensure that the operations do not disclose individual-level data.

DataSHIELD has been successfully used in many large European research projects including LifeCycle (researching the role of novel integrated markers of early-life stressors on health across the lifecycle; Jaddoe et al. (2020), Pinot de Moira et al. (2021)) and ATHLETE (understanding and preventing health effects of environmental hazards and their mixtures; Vrijheid et al. (2021)). It has an ever-expanding set of packages supporting a wide range of analyses, including omics, exposure, mediation, survival and machine learning ( Escriba-Montagut et al. (2024)).

However, a key weakness of DataSHIELD is that it currently lacks effective functionality to perform basic data manipulation, as most developments have focused on extending the analysis capabilities. Many researchers have complained that it is cumbersome to perform basic operations in DataSHIELD, which would normally be straightforward using R. For example, within DataSHIELD, there are currently limited options to (i) recode variables using if-else style operations, (ii) rename variables, (iii) subset columns by column name, (iv) subset rows by multiple conditions, or (v) group data and perform operations by group.

Complicated workarounds are possible, but these greatly increase computational time and lead to verbose analysis scripts. Consider the example of transforming the continuous variable ‘mpg’ (miles per gallon) within the ‘mtcars’ dataset into a 4-level categorical variable (0-15, 15-20, 20-25, >=25). Using the core DataSHIELD package (dsBaseClient), the user is required to first create separate vectors indicating whether participants are above each threshold, which are then added together to create the final variable:

ds. Boole(V1 = “mtcars$mpg”, V2 = 15, Boolean.operator = “>=”, newobj
= “mpg_cat_1”)
ds. Boole(V1 = “mtcars$mpg”, V2 = 20, Boolean.operator = “>=”, newobj
= “mpg_cat_2”)
ds. Boole(V1 = “mtcars$mpg”, V2 = 25, Boolean.operator = “>=”, newobj
= “mpg_cat_3”)
ds.assign (expr = “mpg_cat_1 + mpg_cat_2 + mpg_cat_3”, newobj = “mpg_category”)



In contrast, within R outside DataSHIELD, there are many options for efficient data manipulation. One widely used set of packages is the “Tidyverse,” which comprises a set of packages for data science that share a common design philosophy, grammar and data structures ( Wickham et al. (2019)). These include packages for data manipulation (dplyr), advanced data frames (tibble), and packages for functional programming (purrr) and many others.

Whilst the functionality provided by these packages would greatly improve the user-experience with DataSHIELD, they cannot be used ‘off-the-shelf.’ They first need to be translated into a bespoke DataSHIELD package using the client-server architecture described above, and additional checks need to be written to ensure that they do not inadvertently facilitate the leakage of individual participant data. Here, we report the development of dsTidyverse, a DataSHIELD implementation of selected Tidyverse functions available as free open-source software (LGPLv3) at GitHub and the R CRAN.

## 2. Implementation

### 2.1 Package structure

As described above, each DataSHIELD package contains two components: a client-side and server-side package. The client-side package is installed locally by the researcher and contains functions called in their analysis scripts. The server-side package is installed on the server with the data and contains functions called by the client-side package. For example, to return the mean of a vector, two functions are required: ds.mean() (client-side, included in the dsBaseClient package) and meanDS() (server-side, included in the dsBase package). When an analyst makes a call to ds.mean(), the following steps occur: (i) arguments are checked for validity on the client-side; (ii) an invocation requesting the calling of the function meanDS() is made via the DataSHIELD Interface (DSI) package which handles API calls to the server; (iii) the request, method and arguments are checked for validity on the server-side; (iv) the server-side function meanDS() calculates the mean and performs checks that this value is not disclosive; and (v) the mean of the vector is returned to the client. Following this architecture we implemented two packages: dsTidyverse and dsTidyverseClient. All code was reviewed by co-author SW (an experienced DataSHIELD developer and maintainer of dsBase) to ensure that it met the DataSHIELD disclosure protection standards.

### 2.2 Functionality

Given that DataSHIELD functions need to be implemented individually, it is not realistic to implement the entire set of Tidyverse functions. Instead, we reviewed the existing functionality in DataSHIELD and chose those Tidyverse functions that we believed would significantly improve data manipulation within DataSHIELD. Currently, these functions are from the packages dplyr and tibble, although we are open to adding further functions on request and welcoming Github pull requests. The functions implemented at the time of writing are listed in Table 1.

**Table 1.** Implemented Tidyverse functions.

Package	Function	Description
dplyr	select	Choose columns from a data frame.
dplyr	rename	Rename columns in a data frame.
dplyr	mutate	Create or modify columns.
dplyr	if_else	A vectorised conditional function.
dplyr	case_when	A general vectorised conditional function.
dplyr	bind_cols	Combine data frames by columns.
dplyr	bind_rows	Combine data frames by rows.
dplyr	filter	Filter rows based on conditions.
dplyr	slice	Select rows by position.
dplyr	arrange	Arrange rows by values of a column or multiple columns.
dplyr	group_by	Group data by one or more columns.
dplyr	ungroup	Remove grouping from data.
dplyr	group_keys	Retrieve the group keys from a grouped data frame.
dplyr	distinct	Return unique rows based on certain columns.
tibble	as_tibble	Convert data to a tibble.

dsTidyverse supports non-standard evaluation ( Mailund and Mailund (2018)). The name of the server-side data frame is passed in quotes to df.name, whilst the variable names are passed unquoted and are evaluated as columns within the data frame. Various helper functions can also be used within the ‘tidy_expr’ argument (for example ‘all_of’ and ‘any_of’) to specify multiple variables in filter conditions. See examples at the end of this section on the use of dsTidyverse and the package vignette for a more detailed guide.

### 2.3 Disclosure checks

A key feature of DataSHIELD is the various disclosure checks performed by the server-side package to ensure that individual participant data or any other output that can be used to infer any individual participant information is not returned to the analyst. All but one of the dsTidyverse functions currently implemented are assign-type functions, and these carry a lower risk or direct disclosure, as they do not return anything to the client. However, they carry a risk of indirect exposure, especially in the case of subsetting operations. For example, by creating a subset of data with only one row less than the original data, the summary statistics of the two data frames can be compared to reveal the values of the row in difference. To mitigate against these risks, we implemented the following disclosure checks:

1. We specified a list of permitted functions that can be passed within the ‘tidy_expr’ argument of assign-type functions calls; non-permitted functions will be blocked. The currently permitted functions are:


“everything”, “last_col”, “group_cols”, “starts_with”, “ends_with”, “contains”, “matches”, “num_range”, “all_of”, “any_of”, “where”, “rename”, “mutate”, “if_else”, “case_when”, “mean”, “median”, “mode”, “desc”, “last_col”, “nth”, “where”, “num_range”, “exp”, “sqrt”, “scale”, “round”, “floor”, “ceiling”, “abs”, “sd”, “var”, “sin”, “cos”, “tan”, “asin”, “acos”, “atan”, “c”.

2. We check that the variable names passed within the ‘tidy_expr’ argument are not longer than a specified parameter to reduce the risk of malicious code being passed.
3. To guard against subsetting attacks (malicious attempts to infer individual-level data by taking subsets of data), we check that no subsets are created (e.g. by ds.filter()) with (i) the number of rows lower than a specified parameter or (ii) with the difference between the number of rows of the original dataset and the subset dataset less than a given parameter.
4. We check that the output from ‘ds.group_keys’ (the groups in a grouped data frame) does not contain more groups than a specified parameter relative to the length of the data frame. If no checks were performed this would be highly disclosive, for example if the number of groups was the same as the number of rows, this would return the entire column of participant data.
5. We integrate this package with DataSHIELD disclosure control options that can be set by data owners. This enables data owners to permit or block certain collections of functions depending on the level of privacy security required. For example, dsFilter could be vulnerable to subsetting attacks, so it is blocked in the ‘avocado’ mode (designed to prevent such attacks), but permitted in other privacy modes.

## 3. Examples

To illustrate the improvements brought about by dsTidyverse, we provide three examples using the well-known ‘mtcars’ dataset. Each example contrasts the approach using dsBaseClient with the streamlined alternative using dsTidyverseClient.

### Example 1: Recoding a continuous variable as categorical

We return to the example provided in the introduction, of recoding the continuous variable mpg (miles per gallon) into four fuel efficiency categories:

• 0: <15 (very low)
• 1: 15–20 (low)
• 2: 20–25 (moderate)
• 3: >25 (high)

We previously saw how performing this operation with dsBaseClient was quite verbose. Using dsTidyverseClient, this is achieved in a single call:

ds.case_when(tidy_expr = list (mtcars$mpg < 15 ~ 0, mtcars$mpg >= 15 & mtcars$mpg < 20 ~ 1, mtcars$mpg >= 20 & mtcars$mpg < 25 ~ 2, mtcars$mpg >= 25 ~ 3), newobj = “mpg_category”)



### Example 2: Creating a subset of columns

We want to retain only the columns ‘mpg’, ‘cyl’, ‘hp’, ‘wt’, and ‘gear’. Using dsBaseClient requires identifying column indices and creating a subset:

ds.colnames(“mtcars”) (“mpg”  “cyl”  “disp” “hp”   “drat” “wt”   “qsec” “vs”   “am”   “gear” “carb”)
ds.dataFrameSubset (df.name = “mtcars”, V1 = “id_var”, V2 = “id_var”, Boolean.operator = “==”, keep.cols = c(“1”, “2”, “4”, “6”, “10”), newobj = “subset_mtcars”)



Using dsTidyverseClient, this is greatly simplified:

ds.select (df.name = “mtcars”, tidy_expr = list (mpg, cyl, hp, wt, gear), newobj = “subset_mtcars”)



### Example 3: Filtering on multiple conditions

We create a subset where cars have:

• More than 6 cylinders
• Horsepower greater than 150
• Weight (wt) less than 3.5

Using dsBaseClient, this requires chaining three calls:

ds.dataFrameSubset (df.name = “mtcars”, V1 = “cyl”, V2 = “6”, Boolean.operator = “>”, newobj = “step1”)
ds.dataFrameSubset (df.name = “step1”, V1 = “hp”, V2 = “150”, Boolean.operator = “>”, newobj = “step2”)
ds.dataFrameSubset (df.name = “step2”, V1 = “wt”, V2 = “3.5”, Boolean.operator = “<”, newobj = “filtered_mtcars”)



Using dsTidyverseClient, the same logic is done in one line:

ds.filter (df.name = “mtcars”, tidy_expr = list (cyl > 6 & hp > 150 & wt < 3.5), newobj = “filtered_mtcars”)



These three examples highlight how dsTidyverseClient reduces both code complexity and time investment for common data manipulation tasks. Further use cases and advanced patterns are provided in the package vignette.

## 4. Summary

In this paper we have illustrated the development of dsTidyverseClient, a DataSHIELD implementation of selected tidyverse functions. We hope that this package will provide researchers with more flexible and powerful tools for data manipulation and greatly improve the user experience of DataSHIELD.

## 5. Operation

To use dsTidyverse, the analyst must have:

• R version ≥4.4.0 installed locally
• The dsTidyverseClient package installed from CRAN
• An active DataSHIELD client-server infrastructure with the dsTidyverse package installed on the server side
• An active internet connection and authentication credentials for the federated environment

Full details of setting up DataSHIELD are provided in the DataSHIELD wiki ( https://wiki.datashield.org/en/home).

## Data Availability

No data associated with this article. All vignettes in this paper use the ‘mtcars’ dataset, which is freely available with RStudio. dsTidyverse is maintained as part of the long-running MOLGENIS open-source project for scientific software ( https://molgenis.org/). Requests for the implementation of new functions are welcome, as are contributions from developers. The packages are available to install from https://cran.r-project.org/web/packages/dsTidyverse/index.html and https://cran.r-project.org/web/packages/dsTidyverseClient/index.html Source code is available from: https://github.com/molgenis/dsTidyverse and https://github.com/molgenis/dsTidyverseClient Archived source coded is available at https://doi.org/10.5281/zenodo.15462381. The packages are licensed under LGPLv3.
