# Supplementary material for: ‘dstidyverse’: An Implementation of  TidyverseWithin the DataSHIELD  Ecosystem
Source: F1000Res. 2025 Jun 20;14:606. [Version 1] doi: 10.12688/f1000research.164345.1 (PMC12592851; doi:10.12688/f1000research.164345.1)
Supplement: Peer review history

# Reviewer report: Reviewer response for version 1

**DOI:** 10.5256/f1000research.180842.r419324

**Published:** 2025-11-06

**Authors:** Olaitan I Awe

**Affiliations:** 1 African Society for Bioinformatics and Computational Biology, Cape Town, South Africa

The authors of the manuscript entitled, ‘dstidyverse’: An Implementation of TidyverseWithin the DataSHIELD Ecosystem, described a library or package named dstidyverse which is based on the R programming language. This would make data manipulation easier for users in the DataSHIELD ecosystem. Tidyverse is a popular data wragling library within the R ecosystem and now dsTidyverse implements some of those Tidyverse functions for data manipulation and integration into the DataSHIELD architecture. dstidyverse is open source and freely available and installable from CRAN. I was able to install dsTidyverse in my R version 4.5.0 environment. My minor comment is that the authors should provide examples in their R documentation and the methods should be described with a bit more detail. The package is also available on GitHub (https://github.com/molgenis/ds-tidyverse) thereby making the code findable and reproducible.

Are the conclusions about the tool and its performance adequately supported by the findings presented in the article?

Yes

Is the rationale for developing the new software tool clearly explained?

Yes

Is the description of the software tool technically sound?

Yes

Are sufficient details of the code, methods and analysis (if applicable) provided to allow replication of the software development and its use by others?

Yes

Is sufficient information provided to allow interpretation of the expected output datasets and any results generated using the tool?

Yes

Reviewer Expertise:

Bioinformatics

I confirm that I have read this submission and believe that I have an appropriate level of expertise to confirm that it is of an acceptable scientific standard.

# Reviewer report: Reviewer response for version 1

**DOI:** 10.5256/f1000research.180842.r395741

**Published:** 2025-08-13

**Authors:** Miroslav Puskaric

**Affiliations:** 1 University of Stuttgart, Stuttgart, Germany

This article reports a data manipulation software package dstidyverse, which is already available as a part of the R Data SHIELD library. It addresses the issue of manipulating data such as renaming variables, defining subsets, or conditional formatting, which was prior possible through multiple steps, thus being an overhead for data scientists, even more if analyzing data from multiple sites. 

 Data SHIELD is popular in the health data analysis sector, where there are many use cases. The paper demonstrates the software functionalities on the dataset containing vehicle related information. As a further complement, it would be interested to explore any related work outside of health data.

 A common use case is conducting pooled (federated) analyses across multiple sites, which can be particularly challenging when the data is not harmonized. Describing how this software package facilitates such scenarios would be valuable.

 Many thanks to the authors for the great work, which will further improve workflows for the non-disclosive analysis of sensitive data.

Are the conclusions about the tool and its performance adequately supported by the findings presented in the article?

Yes

Is the rationale for developing the new software tool clearly explained?

Yes

Is the description of the software tool technically sound?

Yes

Are sufficient details of the code, methods and analysis (if applicable) provided to allow replication of the software development and its use by others?

Yes

Is sufficient information provided to allow interpretation of the expected output datasets and any results generated using the tool?

Yes

Reviewer Expertise:

privacy enhancing technologies, management of sensitive data

I confirm that I have read this submission and believe that I have an appropriate level of expertise to confirm that it is of an acceptable scientific standard.
